# Reply: Early onset breast cancer in black British women: a letter to the editor of *British Journal of Cancer* regarding early onset of breast cancer in a group of British black women

**DOI:** 10.1038/sj.bjc.6604337

**Published:** 2008-04-15

**Authors:** R Bowen, S Duffy, D Ryan, I Hart, L Jones

**Affiliations:** 1 Institute of Cancer And Cancer Research UK Clinical Centre, Centre for Tumour Biology Barts and the London Queen Mary's School of Medicine & Dentistry John Vane Science Centre, Charterhouse Square, London EC1M 6BQ, UK

**Sir**,

We thank Mr Dindyal for his interest in our study. He raises some important issues, which highlight the differences between his study and ours.

We detected a substantial difference in the age at presentation between the two ethnic groups. However, due to insufficient age structure information in the population data available, we have been unable to estimate the actual incidence of breast cancer by age and ethnicity.

Unlike African-American studies (Elledge *et al*, 1994; Jatoi *et al*, 2005; Newman *et al*, 2006), we found no difference in breast cancer survival, except in women with small tumours where black women were more than twice as likely to die of their disease. However, by limiting our study to one area of East London, we have been able to control for socioeconomic status. A recent study showed an increased breast cancer mortality in women of West African country of birth (not necessarily black African) domiciled in the UK (Wild *et al*, 2006). This study highlights the difficulties that have existed in this country with regard to assessing the effect of ethnicity on disease.

With regard to the heritage of the women included in our study, all ethnicity data were collected from the hospital's computerised data systems and represent self-reported ethnicity. It was a retrospective study without patient contact and we do not have place of birth recorded for each woman. However, all white women included reported themselves to be white-English, Welsh or Scottish or white-Irish. Anyone defined as white-European or white-Other and those who defined their ethnicity by their religion, for example Jewish, were excluded. The ethnic breakdown of the black women is shown in the table below. None of the self-reporting mixed race was included.


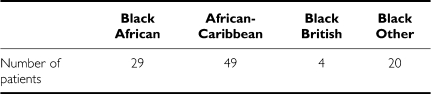


We agree that original heritage may be of importance, especially in light of recent work showing substantial genetic differences between African-American and Jamaican populations (Parra *et al*, 1998), which may also be applicable to the black British populations and have an effect on breast cancer.

We do not have data available for parity and age at first full-term pregnancy, both of which have been shown in some studies to affect risk of breast cancer in African women (Okobia *et al*, 2006). Interestingly, some of the hormonal risk factors associated with breast cancer risk in Caucasian women may not have the same impact in black women (Li *et al*, 2007). We also are not aware of differences in age at first-term pregnancy and breast feeding between our two ethnic groups.

In terms of the implications for screening and treatment, further analytic research at an individual level is indicated, to ascertain, for example, whether screening earlier in life than in the National UK Programme is indicated. In the meantime, women from all ethnic groups should be encouraged to avail themselves of the screening services, and diagnostic and treatment guidelines should be adhered to for patients of all ethnic groups.

Finally, we would agree that further work, with larger studies should be performed to look in detail at breast cancer in other ethnic groups. This was not possible in our referral population due to the lower representation of other ethnic groups in Hackney.
